# Community-engaged optimization of COVID-19 rapid evaluation and testing experiences: roll-out implementation optimization trial

**DOI:** 10.1186/s13012-023-01306-y

**Published:** 2023-10-02

**Authors:** Nicole A. Stadnick, Louise C. Laurent, Kelli L. Cain, Marva Seifert, Maria Linda Burola, Linda Salgin, Paul Watson, William Oswald, Fatima A. Munoz, Sharon F. Velasquez, Justin D. Smith, Jingjing Zou, Borsika A. Rabin

**Affiliations:** 1 University of California San Diego Altman Clinical and Translational Research Institute Dissemination and Implementation Science Center, La Jolla, CA USA; 2 Department of Psychiatry, University of California San Diego, La Jolla, CA USA; 3 Child and Adolescent Services Research Center, San Diego, USA; 4 Department of Obstetrics, Gynecology, and Reproductive Sciences, University of California San Diego, La Jolla, CA USA; 5 Herbert Wertheim School of Public Health and Human Longevity Science, University of California San Diego, La Jolla, CA USA; 6 Department of Medicine, University of California San Diego, La Jolla, CA USA; 7 San Ysidro Health, San Diego, CA USA; 8 Joint Doctoral Program in Public Health School, UC San Diego and San Diego State University, San Diego, CA USA; 9 The Global Action Research Center, San Diego, CA USA; 10 Department of Population Health Sciences, Division of Health System Innovation and Research, Spencer Fox Eccles School of Medicine, University of Utah, Salt Lake City, UT USA

**Keywords:** Implementation science, COVID-19, Underserved communities, Health equity, Promotores, Testing, Roll out implementation optimization design, RE-AIM framework

## Abstract

**Background:**

There continues to be a need for COVID-19 testing that is pragmatic, community-centered, and sustainable. This study will refine and test implementation strategies prioritized by community partners: (1) walk-up no-cost testing, (2) community health worker (promotores)-facilitated testing and preventive care counseling, (3) vending machines that dispense no-cost, self-testing kits.

**Methods:**

A co-designed Theory of Change from an earlier study phase and the Practical, Robust Implementation and Sustainment Model (PRISM) will guide the study design, measures selection, and evaluation. The first aim is to refine and operationalize a multi-component implementation strategy bundle and outcome measures for COVID-19 testing. A Community and Scientific Advisory Board (CSAB) will be established and include community members, clinical providers/staff from the partnering Federally Qualified Health Center (FQHC), public health researchers, policymakers, and a county health department ambassador. Engagement of CSAB members will be assessed through structured ethnography and a survey about the quality and quantity of engagement practices. The second aim is to implement and evaluate the impact of the implementation strategy bundle to optimize COVID-19 testing in communities using a roll-out implementation optimization (ROIO) design. Seven thousand and five hundred community members will be enrolled across four FQHC clinics over 18 months. Participants will be invited to complete an electronic survey about their demographics, health, and COVID-19 testing results and experiences. CSAB members and clinic partners will participate in PRISM fit and determinant assessments prior to each clinic rollout and post-trial. Interviews will be conducted with 60 community participants and 12 providers/staff following a 3-month rollout period at each clinic, inquiring about their experiences with the implementation strategies. Quantitative data will be analyzed using hierarchical multilevel models to determine the impact of implementation strategies. Qualitative data will be analyzed using rapid qualitative approaches to summarize implementation experiences and identify necessary changes prior to subsequent rollouts. A matrix approach will be used to triangulate data from quantitative and qualitative sources based on PRISM domains.

**Discussion:**

This is one of the first pragmatic implementation trials to use a ROIO design and aims to co-create a sustainable and equitable COVID-19 testing program. Findings are likely to generalize to other public health prevention efforts.

**Trial registration:**

NCT05894655 March 2, 2023.

Contributions to the literature

• This study integrates unique and complementary methods from community engagement, implementation science, epidemiology, clinical research, and data science to refine, implement, and sustain a multilevel, multicomponent implementation strategy bundle to optimize COVID-19 testing among underserved communities.
• This study uses a novel roll-out implementation optimization design that balances rigor and responsiveness to the needs of the community and the changing pandemic context to reduce COVID-19 testing disparities.
• This study’s findings have strong potential to generalize to other public health prevention efforts to mitigate health disparities for underserved communities.

## Background

### COVID-19 disparities experienced by underserved communities in California

COVID-19 health inequities abound in low-income, ethnic minority communities. The residents of San Diego who have been most impacted by COVID-19 also experience high rates of poverty, limited educational attainment, are employed in essential and service jobs that require frequent in-person contact, and frequently live in multi-generation households, all of which have exacerbated COVID-19 health disparities with regards to transmission, infection, and negative health sequelae [1]. Throughout the COVID-19 pandemic, rates of COVID-19 have been persistently high in San Diego County’s central and southern communities near the USA/Mexico border.

### Value of community engagement methods to reduce health disparities

Meaningful community engagement is essential to the implementation and sustained use of interventions designed to address health inequities [2, 3]. Community engagement is not a trivial undertaking, especially with underserved communities whose perspectives have historically been excluded from public health planning. Authentic and impactful community engagement requires investment of specialized resources, personnel, and time [4], particularly into activities such as nurturing partnerships with community engagement practitioners to foster relationships between community members and researchers, compensation for community member participation in planning, design and evaluation activities, and interpretation and translation services to encourage equitable participation from individuals in their preferred languages. Community engagement methods vary in their form, intensity, and duration, but typically have a shared goal of “involving communities in decision-making and in the planning, design, governance, and delivery of services; community engagement activities can take many forms, including service user networks, health-care forums, volunteering, or interventions delivered by trained peers” [5].

### Need for development and implementation of strategies for optimal use of COVID-19 testing (evidence-based practice)

The prolonged course of the COVID-19 pandemic, which has been punctuated by several viral variant-driven surges, has illustrated the need for sustainable, long-term, and flexible COVID-19 testing infrastructure and services. Key features of public health testing infrastructure comprise accuracy (including high sensitivity for asymptomatic/pre-symptomatic individuals); temporal and geographic accessibility; low cost; rapid return of results that are easy to understand; and reportability to enable public health agencies to track and respond to changes in the pandemic.

Polymerase chain reaction (PCR) and rapid antigen tests (RATs) are the major COVID-19 testing methods, and they differ markedly in testing features. PCR tests are highly accurate with results reported to clinical providers and public health authorities, with a moderately rapid turnaround time (< 24 h for this study team’s testing lab). However, they are relatively expensive, typically performed at specific and limited locations during certain time windows (often requiring an appointment), and often require insurance coverage and a healthcare provider visit. In contrast, RATs are fast (providing results in 15 min), inexpensive, and can be self-administered using a portable and storable kit. However, they are less sensitive, and results are typically not reported to clinical providers or public health authorities, likely resulting in undercounting of cases. An ideal COVID-19 testing program will take advantage of these complementary testing methods and employ strategies to promote optimal test selection and use.

### Community-driven optimization of COVID-19 testing to reach and engage underserved areas for testing equity (CO-CREATE, phase 1 study)

The current study extends from the CO-CREATE phase 1 study. CO-CREATE was a 2-year study funded through the National Institutes of Health Rapid Acceleration of Diagnostics for Underserved Populations (RADx-UP) initiative. The objective was to understand practices and determinants to uptake of walk-up no-cost COVID-19 testing for underserved communities in partnership with a federally qualified health center (FQHC) near the US/Mexico border. In the phase 1 study, a 22-member Community and Scientific Advisory Board (CSAB) was established and met monthly to participate in an Appreciative Inquiry method led by the Global Action Research Center (ARC), a community partner and social change organization. The findings from this co-creation process were implemented at a walk-up no-cost COVID-19 testing site, which resulted in the administration of 24,422 tests performed (> 13,253 unique participants) over a 2-year timeframe. Details about the phase 1 study and CSAB are reported elsewhere [6–8].

### Implementation strategies

The current study will refine and test three implementation strategies for optimal COVID-19 testing (evidence-based practice): (1) walk-up no-cost testing, (2) Community Health Worker (Promotores)-facilitated testing and preventive care counseling, (3) vending machines that dispense no-cost, self-testing kits. These strategies were prioritized by the CSAB of the phase 1 study using implementation mapping [9].

#### Walk-up no-cost testing protocol

A no-appointment, walk-up point-of-care rapid testing at the partnering FQHC will continue at clinic 1 based on the success of the phase 1 study. Testing will be offered several times per week and titrated to lower intensity following the first clinic rollout. Trained, bilingual staff will oversee the on-site testing protocol. The walk-up testing protocol will not be offered at the other clinic sites.

#### Promotora-facilitated testing and preventive care counseling

Trained, bilingual promotores will provide health counseling on these topics: when COVID-19 testing is recommended; which test to use; testing instructions; and how to interpret the results. Promotores will also offer general preventive care reminders, such as flu shots, blood pressure checks, and hemoglobin A1c screenings. Promotores will be available full-time during clinic working hours throughout the first 3 months of roll-out at each clinic and subsequently available on an as-needed basis.

#### Vending machines that dispense no-cost self-testing kits

Vending machines located outside of clinics will provide interactive instructions in Spanish and English for accessing no-cost self-administered COVID-19 test kits. The vending machines will be available 7 days/week and will be accessible to the public. The vending machine locations will be identified with guidance from the partner FQHC to consider potential workflow and service implications. Study registration and consent will be required to obtain a test kit from the vending machines. Alternative sources for testing kits will be provided for individuals who do not want to register and consent to the study.

### Theory of change and implementation science framework

This study is guided by a Theory of Change and the Practical, Robust Implementation and Sustainability Model.

#### Theory of change (ToC)

A ToC can be used to guide the planning, implementation, and evaluation of public health programs in low-resource settings and provides a comprehensive illustration of how and why a desired change is expected to happen in a particular context [10, 11]. In the phase 1 study, a rigorous, multi-step process was used with the CSAB to create a ToC [6] that will be confirmed and used to inform implementation and evaluation planning. This ToC illustrates the root causes, conditions needed, and measures of success to mitigate disparities in COVID-19 testing access and care.

#### Practical, robust implementation and sustainability model (PRISM)

PRISM will guide the optimization, implementation, and evaluation of the study’s multi-component implementation strategy bundle [12–14]. PRISM allows for multi-level conceptualization of implementation efforts (context domains) and provides guidance on how to measure relevant implementation outcomes through integration of the RE-AIM measures (Reach, Effectiveness, Adoption, Implementation, Maintenance)—one of the most widely used set of implementation measures [15]. PRISM is ideal for guiding real-world community-based research because it accounts for multi-level effects, it builds on several implementation science frameworks, and it can guide development, implementation, and evaluation. PRISM will be used to consider multiple levels of context, recipient characteristics (community members and promotores, conceptualized as delivery agents), intervention characteristics (as perceived by diverse partners), implementation and sustainability infrastructure within communities and clinics, and the external environment (national guidelines and regulations regarding COVID-19 testing and management). In addition to effectiveness, the study will assess the RE-AIM outcomes of reach, adoption, implementation, and maintenance.

### Aims

The specific aims of the study are as follows:

#### Aim 1

To refine and operationalize a multi-component implementation strategy bundle and a related set of outcome metrics for COVID-19 testing.

#### Aim 2

To implement and evaluate the impact of the innovative, multilevel, and multicomponent implementation strategy bundle to optimize COVID-19 rapid testing among underserved communities in Central and South San Diego using a roll-out implementation optimization study design across 4 clinics over 18 months.

## Methods

### Study design

**Fig. 1:**
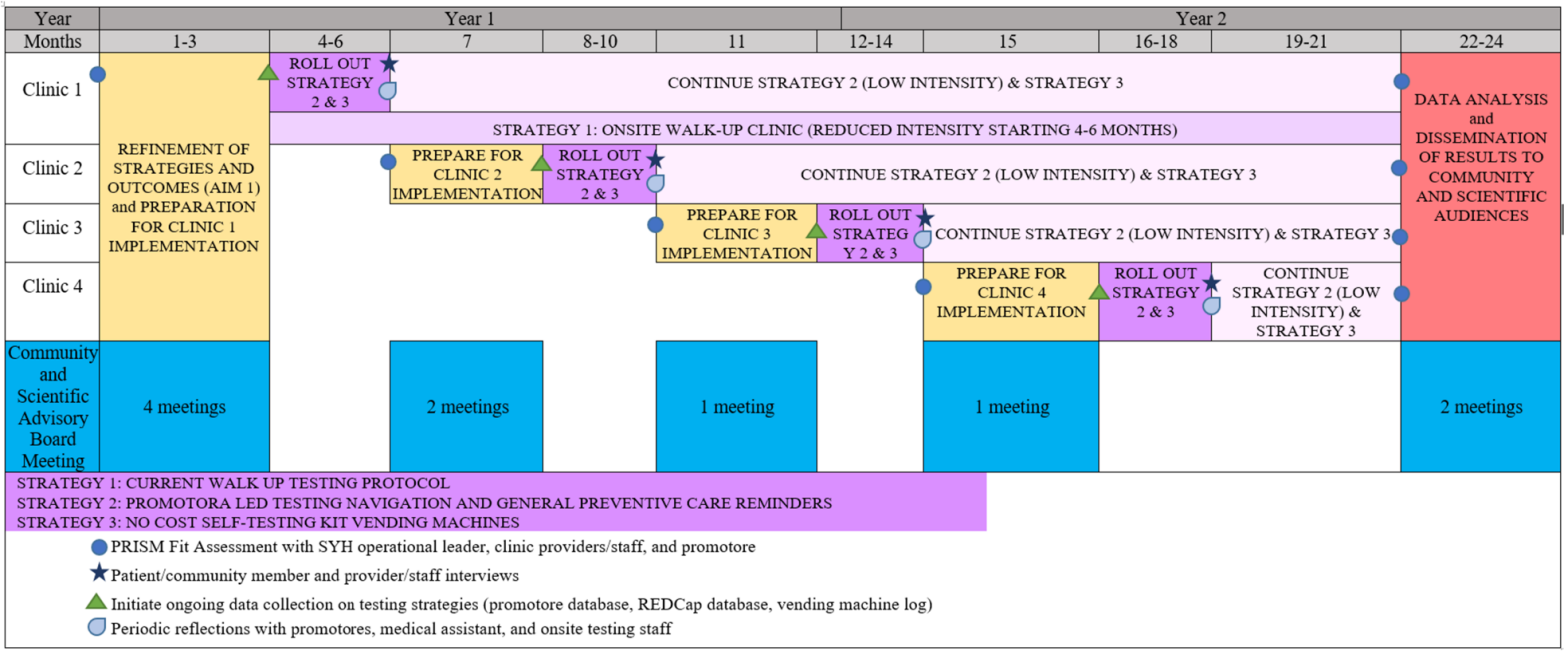
CO-CREATE-Ex participatory (Aim 1) and ROIO (Aim 2) design and timeline

The multi-component COVID-19 testing implementation strategy bundle and outcome metrics will be operationalized and refined using a participatory approach engaging diverse community, clinic, and research partners through a CSAB, brainwriting premortem interviews [16] and the PRISM Fit Assessment (Aim 1). The implementation strategy bundle to optimize COVID-19 testing among underserved communities will be tested using a roll-out implementation optimization (ROIO) study design [17] (Aim 2). See Fig. 1 for the study design and timeline.

The primary outcome is defined as offering the most clinically useful COVID-19 test and returning results within an optimal timeframe to enable appropriate treatment and decrease transmission, in the most acceptable, timely, and feasible manner. Secondary outcomes include increased: reach to priority communities; adoption and meaningful, sustained implementation of the testing strategy bundle; optimal COVID-19 testing experiences; and metrics that matter most to clinical and community partners such as rates of preventive care engagement. The ROIO study design allows for iterative refinement of the implementation strategies after each clinic roll-out [17]. Longitudinal data mixed-methods data collection and analysis with multiple partners and community members will inform necessary refinements and assessment of the impact of the strategy bundle on key outcomes over time.

### Setting

This study will be conducted in partnership with a high-performing FQHC that serves low-income and uninsured San Diegans and has a long-standing relationship with the lead university. For this study, we will focus on clinics in Central and South San Diego. As shown in Table 1, the 4 target clinics serve primarily Latino and Black patients, and a large proportion report Spanish as their primary language. Preventive healthcare rates for commonly recommended adult preventive screenings and immunizations are low, particularly for blood pressure (*M* = 60.6% across clinics) and flu immunization (*M* = 20.4% across clinics) [18].

**Table 1:** FQHC clinic characteristics

	Clinic 1	Clinic 2	Clinic 3	Clinic 4
Total # patients	18,124	20,102	8328	8120
Patient race/ethnicity				
Latino	91.6%	79.0%	61.2%	74.3%
Black	1.3%	3.0%	16.9%	12.1%
Asian	1.5%	4.7%	4.7%	2.2%
White	14.4%	15.0%	29.4%	15.3%
Patient preferred language				
Spanish	70.1%	56.7%	36.0%	48.8%
Adult preventive health services (% of “active” patients who completed a medical visit and had a preventive health service in the past 18 months)				
Blood pressure screening	55.4%	62.3%	58.2%	66.3%
HbA1c screening	78.9%	79.3%	72.8%	81.6%
Flu immunization	25.0%	15.5%	16.2%	24.9%
COVID-19 cases/100,000 (2/20–4/22)	48,219	31,623	34,209	35,716
RATs distributed since 1/22	785 (*M* = 196/month)	499 (*M* = 125/month)	244 (*M* = 61/month)	192 (*M* = 48/month)

### Participants

Recruitment of community members will occur at the FQHC clinics through collaborative efforts between promotores, and on-site research staff. Recruitment and outreach will occur on-site for patients coming to the clinic for appointments, through outreach at local community gathering locations, phone calls, and/or emails to existing FQHC patients, outreach by CSAB members utilizing their existing networks, social media posts, flyers, and/or billboards. It is anticipated that participants will represent the ethnic, racial, and sex distributions of the registered patients at each participating clinic. Promotores will be hired by the partnering FQHC and speak the languages that are most common in the local communities and clinics (Spanish and English).

### Community and scientific advisory board (CSAB)

Building on the phase 1 CSAB, a slightly modified membership for the current study’s CSAB will facilitate representation from communities around the 4 participating clinics for the Aim 2 implementation and evaluation activities. In addition to 4 community partners, the CSAB will include 2–3 clinical staff, 1–2 public health researchers, 1–2 policy partners, and a County Public Health Department ambassador. CSAB community partners will be selected based on their role as community weavers—that is, individuals with lived experience as a member of an underserved community who are cultural brokers between communities, health systems, and researchers to co-create community-driven public health solutions.

### Aim 1 procedures

To refine and operationalize the implementation strategy bundle and measures of implementation and sustainment success, several participatory methods will be used. During CSAB meetings led by the Global ARC, initial strategies, measures, and data collection plans will be presented. The research team will facilitate group brainwriting premortem interviews [16] with the CSAB to gather information about what specific components of the implementation strategy bundle and measurement might be prone to fail and to co-create solutions to prevent these failures.

#### Data collection and analysis

Aim 1 data collection will focus on evaluating the quality, extent, and content of partner engagement through the participatory CSAB activities. An established multimethod ethnographic approach [19] using a refined documentation form will be completed by trained research interns at each CSAB meeting, and a partner engagement survey based on validated measure from Goodman and colleagues [20] will be completed by all CSAB attendees after each session. Data from the partner engagement survey will be analyzed after each CSAB meeting to inform post-meeting debriefings with the Global ARC and research team. Aim 1 data (brainwriting premortem interviews, ethnographic documentation forms, engagement surveys) will be analyzed using a rapid qualitative analytic approach and simple descriptive statistics. Summarized data from these sources will be triangulate using a joint display analysis [21, 22]. This methodology has been described in more detail in Rabin et al. [19]. In addition, the research team will document adaptations made to the implementation strategies throughout the Aim 1 refinement process (and throughout Aim 2) using methods developed by Rabin et al. and that was used in the phase 1 study [19, 23].

### Aim 2 procedures

An innovative ROIO study design [17] will be used to implement and evaluate the multilevel, and multicomponent implementation strategy bundle (Fig. 1). Four cycles of preparation, initial roll-out (3 months), and sustainment will be launched over 21 months. Refinements to the implementation strategy bundle and processes will be made prior to each new roll-out based on information collected from the prior roll-out (or the CSAB preparation for Clinic 1) and a PRISM Fit Assessment. The PRISM Fit Assessment will explore how well the proposed strategy bundle aligns with the multi-level context of Clinic 1, how likely the proposed bundle will lead to increased rapid COVID-19 testing, reach of community members, and adoption and sustained implementation. The PRISM Fit Assessment will be repeated prior to each subsequent clinic roll-out. Each PRISM Fit assessment will involve clinic staff, promotores, and clinic operational leaders completing a 21-item survey developed around the key PRISM context domains and related RE-AIM outcomes independently followed by a group review of the summary results to discuss any misalignments and additional needs for refinement in the context of the specific clinic. This process will be repeated for each clinic prior to roll-out and at the end of the study period.

Preparation phases last 3 months and include data collection from patients/community members, clinic providers/staff, and promotores, medical assistant, and onsite testing staff, rapid analysis of this information and triangulation with other testing information, presentation to and refinement with the CSAB, and finalization based on PRISM Fit Assessment data. During the initial roll-out phase (3 months), the full-intensity implementation strategy bundle is provided at the clinic. After 3 months, clinics move into continued services with vending machines and promotores, available on an as-needed basis. Walk-up testing will also decrease in intensity moving to a setup with one part-time staff operating the walk-up testing.

#### Data collection

Quantitative and qualitative data will be collected using multiple databases and methods. Data collection methods are summarized in Table 2 with the data source name, type of data collected, origin of data and timing for data collection, and associated PRISM domains assessed. Data from the PRISM Fit Assessment will be summarized as descriptive statistics and displayed in the form of bar graphs to inform the group action planning discussion. Key implementation and clinical and public health effectiveness outcomes are summarized in Table 3. The timing for each data collection method is also noted in Fig. 1. Participants will be required to provide contact information and minimum symptom and exposure data prior to obtaining rapid tests and the type of test and timing of testing will be captured for both the walk-up onsite testing and vending machine testing along with basic demographics. Participants will also be invited to provide rapid test results and receive personalized health counseling based on their test result.

**Table 2:** Data sources, data types, origin and PRISM domains

Data source name	Type	Origin/timing	PRISM domains
REDCap study database for rapid COVID-19 testing (QUAL + QUANT)	Database completed by walk-up onsite testing staff about number of tests completed, reason and timing for testing including symptoms and exposure, community member demographics	Walk-up onsite testing staff Ongoing	Reach Implementation Maintenance
Preventive care status and health characteristics (QUANT)	Electronic health records data on primary and secondary outcomes, participant demographics, other health characteristics	SYH Research Assistant accessing electronic health records After each initial roll-out	Reach Recipient characteristics Effectiveness
Promotore support activities database (QUAL + QUANT)	# of days and hours promotore services are provided; # and type of support activities	Promotores Ongoing	Adoption Implementation Maintenance
Vending machine automatic data log (QUANT)	#, timing, type of interactions with vending machine; reason and timing for testing, type of test used; participant demographics	Programmed in vending machine Ongoing	Reach Effectiveness Implementation Maintenance
Periodic reflections with promotores, medical assistant, and onsite testing staff (QUAL)	Key lessons learned, facilitators, challenges of implementation of the testing strategy bundle	Promotore FQHC Research Assistant Other onsite testing staff After each initial roll-out	Intervention characteristics Implementation and sustainability infrastructure External environment
Community member interviews (QUAL)	Experience with the multicomponent implementation strategy for testing at each clinic	Subset of participants (*n* = 60, 15/clinic) After each initial roll-out	Implementation Effectiveness Maintenance
FQHC provider and staff interviews (QUAL)	Experience with the multicomponent implementation strategy for testing at each clinic	Subset of providers and staff (*n* = 12, 3/clinic) After each initial roll-out	Recipient characteristics Adoption
PRISM Fit Assessment (QUAL + QUANT)	Fit of proposed strategy bundle with multilevel PRISM context domains and RE-AIM outcomes with clinic operational leaders, providers and staff, and promotores	Clinic operational leaders, providers and staff, promotores Before each roll-out and at the end of the study period	All PRISM context and RE-AIM outcomes
Adaptation documentation database (QUAL + QUANT)	Ongoing documentation of adaptations occurring in Aim 1 and Aim 2 to implementation strategies and procedures	UCSD clinical research staff Ongoing	Implementation

**Table 3:** Implementation and effectiveness outcomes

Implementation outcomes	Reach	The absolute number, proportion, and representativeness of community members who attend the walk-up onsite testing; interact with the promotores for support; and/or utilize the vending machines compared to all eligible community members on key characteristics. Reasons for non-participation
	Adoption	Demographic and professional characteristics of promotores (adoption staff level) and characteristics of clinics (adoption site level)
	Implementation	# of days and hours walk-up onsite testing is delivered, # of staff at testing, # of test completed at walk-up onsite testing, # of days and hours promotores delivering support per clinic and the type of support delivered, # of days and hours vending machines operate at clinic site and types of services provided through the machine, number of tests dispensed through the vending machines
	Maintenance	Ongoing operation of walk-up onsite testing beyond initial roll-out at Clinic 1; ongoing promotore services provided beyond initial roll-out (first 3 months) at all clinics (and type of support); ongoing operation of vending machines beyond initial roll-out (first 3 months)
Effectiveness (clinical and public health) outcomes	COVID-19 RAT completion	Number of RATs distributed through the walk-up onsite testing and vending machines
	Optimal COVID-19 testing experience	Offering the most clinically useful COVID-19 test and returning results within an optimal timeframe to enable appropriate treatment and decrease transmission, in the most acceptable, timely, and feasible manner
	Preventive care engagement	Up-to-date age/sex-specific preventive care (e.g., flu shot; cancer screening) based on participants’ demographic and health characteristics

Qualitative interviews with patients/community members and clinic providers/staff will be conducted after each initial roll-out period (after 3 months of implementation) to understand their experiences with the implementation strategy bundle and identify potential areas for improvement. A total of 60 interviews with patients/community members evenly recruited from each of the 4 clinics will be conducted. Patients/community members will be identified for interviews based on their interest expressed when initially obtaining tests as part of the walk-in onsite clinic or vending machine. An additional set of interviews will be conducted with 12 providers/staff (3/clinic) identified based on their involvement in supporting the implementation or having exposure to the implementation strategy bundle.

#### Data analysis

Quantitative data from study databases will be summarized using simple descriptive statistics including frequencies, measures of central tendency, and variability along with data visualization methods, such as frequency tables, bar charts, line graphs, and scatter plots to understand patterns and characteristics of testing uptake, exposures, and symptoms and to guide adaptive implementation. Missingness in data will be examined via sensitivity analysis and imputation methods will be applied when appropriate. Parametric and non-parametric tests will be employed to compare the survey responses between the clinics while controlling for a variety of demographic and socioeconomic variables, including the FQHC clinic cluster. Data collected at the point of test distribution (collected by study coordinators, promotores, and vending machines) will be used to evaluate implementation strategy effectiveness on testing uptake. Hierarchical multilevel models will be used to ensure the inclusion of random effects, variation between clusters, variation between times within clusters, and the fixed effect of time which will be estimated independently of treatment effect so that systematic change over time will not be mistaken for effect of treatment. This is key, based on significant variations in testing uptake observed during previous COVID-19 variant waves. To account for potential within-clinic correlation, data will be reduced for each cluster to a single observation, and then subject to standard two-sample analyses. Secondary analyses will be conducted to determine the appropriateness of the test type distribution within the context of symptom onset and exposure time frame.

Rapid matrix analysis of patient/community member and provider/staff interviews and periodic reflections will be conducted to identify key concerns and opportunities for refinements. This information, along with data about the implementation of the strategy bundle and testing results, will be summarized and presented to the CSAB (two sessions after Clinic 1 roll-out and one session after each consecutive clinic roll-out). A list of possible refinements will be identified based on input from the CSAB and final decisions about refinements will be made by the community-clinical-research team. Any changes made to the strategy bundle and procedures will be documented in the Adaptation documentation database. After refinements to the multi-component strategy bundle have been completed, the PRISM Fit Assessment will be conducted with the next clinic to allow for clinic-specific further adjustments. A matrix approach will be used to triangulate data from quantitative and qualitative sources using the key domains of PRISM. A joint display will also be produced to support the integration of data sources [21, 22].

### Power size calculations

**Fig. 2:**
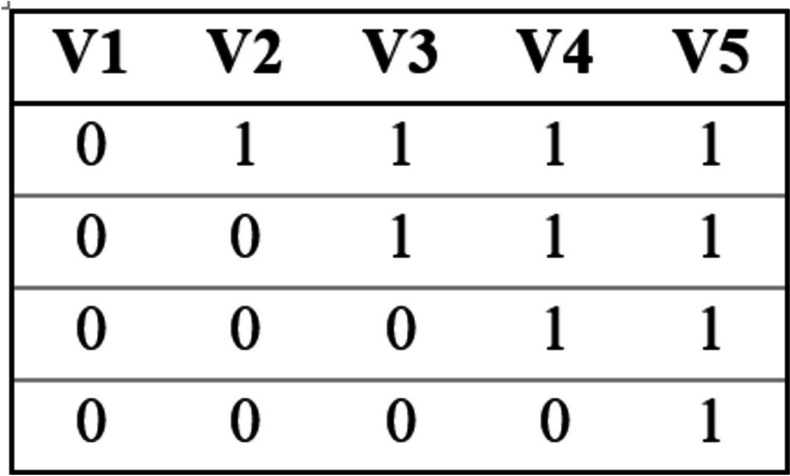
Design matrix structure: one cluster per treatment sequence

The number of participating clusters/clinics and target clinic participant enrollment for the ROIO study design was determined using cluster randomized trial sample size estimation tools. Per the ROIO study design, each clinic cluster will begin in the control group and then cross over into the intervention group at different time points and then continue with the intervention until the end of the study (see Fig. 2). Although it is anticipated that the implementation strategies as outlined by CSAB will undergo some adjustments during the implementation process, the target cluster/clinic sample size estimates are based on a stepped wedge design using serial cross-sectional evaluations of electronic medical records and test kits distributed. It is estimated that the study will have the capacity to distribute 52,000 FDA-authorized rapid antigen tests provided by the San Diego County Health and Human Services and the California Department of Public Health. The primary outcome is the increased distribution of RATs within the communities near each of the 4 clinics. This will be measured using a proxy outcome defined as the proportion of clinic patients visits to the number of RAT kits distributed per month at each participating clinic. Currently, RAT kit distribution among target clinic patients ranges from 7 to 13% (*M* = 9%) with approximately 430 kits being distributed per month between all four clinics. The aim is to increase distribution of test kits to 40% of clinic patient encounters per month, which would result in the distribution of 1822 kits per month during the final treatment sequence. Based on data collected from the phase 1 study, approximately 40% of testing uptake occurred among clinic patients and the remaining 60% from community members even when the intervention was clinic-based. It is anticipated that there will be a similar community uptake during this study. Based on the sequential rollout outlined in the Fig. 2 with rollout for Clinic 1 at month 4, Clinic 2 at month 8, Clinic 3 at month 12, and Clinic 4 at month 16, over the course of the entire study it is anticipated that a total distribution of 20,503 tests, assuming average distribution per clinic. Estimating that this will equal approximately 40% of total increase and the remaining uptake to occur among community members, a total uptake of 58,091 test kits is estimated over the course of the study. Using the Shiny CRT Calculator [24], it was determined that a minimum cluster sample size of 285 across 4 clinics will provide sufficient power (beta > 0.8) to determine an intervention proportion increase of 0.05 to 0.25 assuming the following: stepped-wedge design, cross-sectional sampling structure at each of 4 sequences (approximately every 3 months in the study), 0.5 coefficient of variation in cluster sizes (based on average number of patient encounters per month per clinic), intra-cluster correlation (ICC) of 0.02, for our binary outcome (test or no-test). It was also assumed that there will be an exchangeable cluster correlation structure as SARS-CoV-2 testing demand has been impacted significantly by changes in variant severity and transmissibility.

## Discussion

This study capitalizes on the unique and complementary methods from community engagement, implementation science, epidemiology, clinical research, and data science to refine, implement, and sustain a multilevel, multicomponent implementation strategy bundle to optimize COVID-19 rapid testing among underserved communities. This study will use a ROIO design to iteratively refine implementation in response to dynamic public health policy and healthcare delivery contexts. This is one of the first pragmatic implementation trials to use an ROIO design, affording opportunities to further develop best practices for adaptive implementation science study designs. Further, although this study prioritizes focus on COVID-19 testing as the evidence-based practice vehicle, the design and methods used can easily be transferred to other public health prevention programs such as sexually transmitted infection testing, flu testing, and viral panels.

Balanced with these strengths are potential challenges and limitations. One challenge will be balancing reach with contextual considerations and competing priorities. Meaningful community engagement to sustainably reduce health disparities requires expertise from community, clinical, and research partners as well as sufficient material resources. The research team has collaborated with the study’s community and clinical partners throughout each step in the phase 1 study and the current study to ensure adherence to rigorous, acceptable, and capacity-building approaches. However, competing organizational and policy priorities may impact study activities. These will be documented in the adaptations tracking form and included in all relevant study analyses. In addition, the research team will rely on the decades of community-engaged participatory action research led by the Global ARC and the quality care and clinical research supported by the partnering FQHC to reduce health disparities in their patient population. The study team will leverage ongoing, weekly meetings to assess any study challenges that arise and jointly make decisions to resolve them. Another limitation is that randomization of clinics or community members will not be possible. Given the duration of the study (2 years), it was only feasible to include 4 clinics with one clinic being the original clinic that was the host of the phase 1 study. Due to the pragmatic and organizational requirements of the FQHC, the clinic roll-out order will be sequenced in a non-randomized fashion, but potential confounding clinic-level and participant-level variables will be collected and included in multilevel models to contextualize findings.

This study has the potential to make a substantial contribution to the field of implementation science through the application of the newly described ROIO design, which will rigorously allow for a flexible and sustainable approach that promotes responsiveness to both the needs of the community and the changing pandemic context while reducing COVID-19 testing disparities. In addition, this study will advance the call for action [25] to explicitly examine the role of implementation vis-à-vis implementation strategies and other health outcomes. It is expected that the study will exert a significant public health impact by increasing optimal COVID-19 testing for unserved or underserved communities near the US/Mexico border.

## Data Availability

Not applicable.

## Abbreviations

CSAB: Community and Scientific Advisory Board
FDA: Federal Drug Administration
FQHC: Federally Qualified Health Center
Global ARC: Global Action Research Center
PCR: Polymerase Chain Reaction
PRISM: Practical, Robust Implementation and Sustainability Model
QUANT: Quantitative
QUAL: Qualitative
RAT: Rapid Antigen Test
RE-AIM: Reach, Effectiveness, Adoption, Implementation, and Maintenance
REDCap: Research Electronic Data Capture
ROIO: Roll-Out Implementation Optimization
ToC: Theory of Change
